# Characterization and phylogenetic analysis of the mitochondrial genome of *Rhinogobius wuyiensis* Li & Zhong, 2007 (Gobiiformes: Gobiidae: Gobionellinae)

**DOI:** 10.1080/23802359.2025.2466584

**Published:** 2025-02-18

**Authors:** Xing Peng Han, Lin Song, Quan Wang, Xiao Jiang Chen

**Affiliations:** College of Fisheries Science and Technology, Jiangsu Agri-animal Husbandry Vocational College, Taizhou, PR China

**Keywords:** *Rhinogobius wuyiensis*, mitogenome, phylogenetic analysis

## Abstract

The mitochondrial genome of *Rhinogobius wuyiensis* Li & Zhong, 2007 was initially assembled using sequencing data from the Illumina HiSeq platform (San Diego, CA), resulting in a circular mitogenome of 16,502 base pairs with a pronounced A + T nucleotide bias. Maximum-likelihood (ML) and Bayesian inference (BI) revealed the phylogenetic relationships within the genus *Rhinogobius*, indicating that *R. wuyiensis* is closely related to a clade comprising *R. maculagenys*, *R. shennongensis*, *R. niger*, and *R. lentiginis*. This study enriches genetic resources and advances the understanding of phylogenetics within the genus *Rhinogobius*.

## 1. Introduction

The genus *Rhinogobius* Gill 1859 is widely distributed across East and Southeast Asia. Known for its remarkable diversity, *Rhinogobius* encompasses over 100 recognized species, many of which await formal taxonomic description (Hu et al. 2022). These small benthic fishes occupy diverse aquatic habitats, including swiftly flowing streams, stagnant ponds, and estuarine environments across their native range.

Although *Rhinogobius* species have negligible commercial value in fisheries, they hold some ornamental value in the pet trade. Recent surveys indicate that approximately 30 *Rhinogobius* species are circulating within the Chinese market (Liu et al. 2021). Furthermore, *Rhinogobius* species have been utilized for environmental monitoring (Sadeghi et al. 2019), assessing aquatic ecosystem health, studying ecological diversity, and analyzing biogeographic patterns (Ohara et al. 2008; Liao et al. 2021). The proliferation of hydraulic facilities has led to considerable habitat fragmentation within fish populations, making *Rhinogobius* species an ideal model for investigating the impacts of this fragmentation on fish genetic diversity (Ding et al. 2020).

Existing research on *Rhinogobius wuyiensis* Li & Zhong 2007 is extremely limited, and no relevant molecular data are available in GenBank. This study aims to provide the first molecular data for *R. wuyiensis* and to conduct a phylogenetic analysis within the *Rhinogobius* genus.

## 2. Materials and methods

### 2.1. Materials

**Figure 1.**
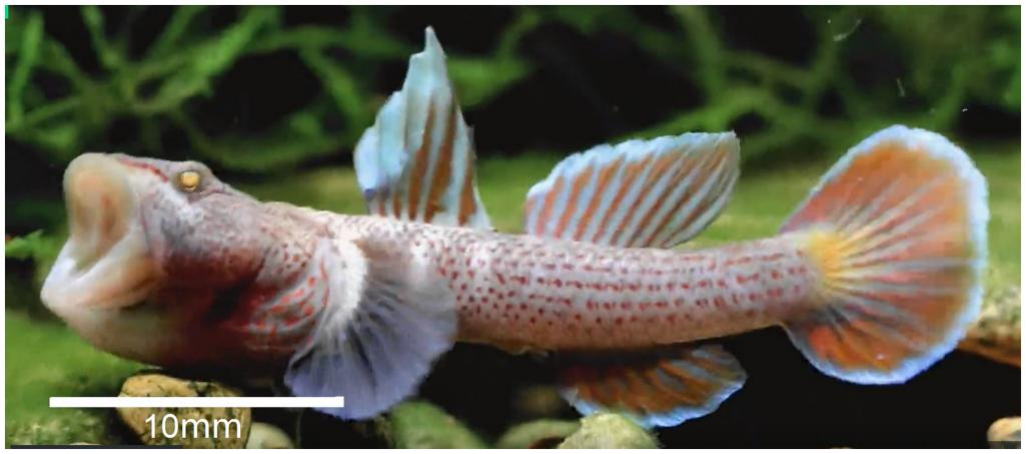
Collection site of *R. wuyiensis* in the Wuyi River, Zhejiang Province, China (28°57′ 58.129″ N, 119°47′ 27.539″ E) (photo by Xiao Jiang Chen on 20 September 2021).

In August 2021, samples of *R. wuyiensis* were collected from the Wuyi River (28°57′ 58.129″ N, 119°47′ 27.539″ E) in Zhejiang Province, China (Figure 1). The diagnostic characteristics of *R. wuyiensis* are as follows: dorsal fin VI, I-8-9; anal fin I-7-9; 15–18 pectoral fins; abdominal fin I-5; caudal fin formula 16–18; 30–31 longitudinal scales; 9–11 transverse scales; dorsal fin anterior scales 0–4; and vertebral count 10 + 16 = 26 (Li and Zhong 2007). *R. wuyiensis* exhibits head spot patterns similar to *R. lentiginis* but differs in: having head cephalic lateralis canals (absent in *R. lentiginis*); a vertebral count of 10 + 16 = 26 (vs. 27); an anal fin with 7–9 branched rays (vs. 6–7); irregular head spots (vs. regular); and male inner branchiostegal membrane without dots (vs. many dots) (Li and Zhong 2007).

During sample collection, the status of the specimens (alive or dead) was recorded. Dead specimens were immediately transferred to 95% ethanol for long-term storage at the Fish Specimen Collections of the College of Aquatic Science and Technology (Voucher ID: ASTIH-21b1108d20, Xiao Jiang Chen, cq_cxj@126.com). Live specimens were euthanized by immersion in a clove oil solution (100 mg/L) and preserved in 95% ethanol at −20 °C until DNA extraction and further analysis.

### 2.2. Methods

Genomic DNA was extracted from muscle tissue using the TGuide Cell/Tissue Genomic DNA Extraction Kit (OSR-M401; Tiangen, Beijing, China). Quality control measures for sample concentration, purity (260/280 and 260/230 ratios), and integrity were conducted using a NanoDrop 2000 spectrophotometer (Thermo Fisher Scientific, Waltham, MA). A DNA library, including sheared mitochondrial DNA, was prepared using the MGIEasy DNA Library Prep Kit (MGI Technology, Shenzhen, China). Purification and size selection of the library were performed with Agencourt SPRIselect (Beckman Coulter, Brea, CA), and the library quality was assessed with an Agilent 2100 Bioanalyzer (Agilent Technologies, Santa Clara, CA). Sequencing was conducted on the Illumina HiSeq 4000 platform (Illumina, San Diego, CA) with 150-bp paired-end reads.

Quality assessment of sequencing reads was conducted using FastQC (http://www.bioinformatics.babraham.ac.uk/projects/fastqc). Low-quality reads and adapter sequences were removed with Trimmomatic v0.40 (Bolger et al. 2014). Clean reads were aligned to the reference mitochondrial genome of *Rhinogobius brunneus* (LC648312) using the Burrows–Wheeler Aligner (BWA) v0.7.17 (Li and Durbin 2009). Aligned mitochondrial reads were extracted using Samtools v1.9 (Li and Durbin 2009) and assembled with MetaSPAdes v3.13.0 (Nurk et al. 2017). The preliminary assembly results indicated a maximum depth coverage of 16,629×, minimum depth coverage of 500×, and an average depth coverage of 724.75×. The sequencing depth and coverage map of *R. wuyiensis* mitochondrial genome was presented in Supplementary material (Figure S1). Complete mitochondrial genome annotation was performed using the MitoFish webserver (http://mitofish.aori.u-tokyo.ac.jp/, Iwasaki et al. 2013), and the mitogenome map was generated with Proksee (https://proksee.ca/, Grant and Stothard 2008).

To avoid data redundancy, a total of 23 mitochondrial genomes from the genus *Rhinogobius* were retrieved from the NCBI database using BLAST. The datasets included nucleotide sequences of all 13 protein-coding genes (PCGs), which were used to construct phylogenetic trees with *Tridentiger obscurus* (MF663787.1) from the subfamily Gobionellinae as the outgroup (Table 1). The multiple sequence alignment was performed using the MUSCLE algorithm in MEGA X (Kumar et al. 2018) with default settings. Poorly aligned regions were removed using the Gblocks 0.91b web server (Castresana 2000) with the setting ‘Do not allow many contiguous non-conserved positions’. Genetic distances were subsequently computed using the P-distance model in MEGA X. The optimal substitution model, identified by both jModelTest v2.1.10 (Darriba et al. 2012) and the MEGA X analysis, was GTR + G + I. Maximum-likelihood (ML) phylogenetic trees were constructed in MEGA X with 1000 bootstrap replicates to assess node support. Bayesian inference (BI) of phylogeny was performed using MrBayes v3.2.7a (Ronquist et al. 2012), with two parallel runs of four Markov chains for 2,000,000 generations. The initial 25% of trees were discarded as burn-in, and stationarity was assumed once the average standard deviation of split frequencies dropped below 0.01.

**Table 1.** The taxonomic nomenclature, GenBank accession identifiers, and literature citations for all 25 mitochondrial genomes used in constructing the phylogenetic tree.

Family Subfamily	Genus	Species name	Accession number	Reference
Gobiidae Gobionellinae	*Rhinogobius*	*Rhinogobius nagoyae*	LC648316.1	Maeda et al. (2021)
		*Rhinogobius sp. MO*	LC648313.1	Maeda et al. (2021)
		*Rhinogobius brunneus*	LC648312.1	Maeda et al. (2021)
		*Rhinogobius yaima*	LC648308.1	Maeda et al. (2021)
		*Rhinogobius yonezawai*	LC648310.1	Maeda et al. (2021)
		*Rhinogobius flumineus*	LC648306.1	Maeda et al. (2021)
		*Rhinogobius formosanus*	MT363639.1	Yang et al. (2020)
		*Rhinogobius rubromaculatus*	KU674802.1	–
		*Rhinogobius szechuanensis*	OM617727.1	Liu et al. (2023)
		*Rhinogobius leavelli*	MH729000.1	Zhang and Shen (2019)
		*Rhinogobius davidi*	OM617724.1	Song et al. (2023)
		*Rhinogobius cliffordpopei*	MK288030.1	Chen et al. (2019)
		*Rhinogobius wuyiensis*	OM678441.1	Our paper
		*Rhinogobius lentiginis*	OM617725.1	Song et al. (2023)
		*Rhinogobius niger*	OM791349.1	–
		*Rhinogobius shennongensis*	OM961050.1	Shang et al. (2022)
		*Rhinogobius maculagenys*	OK545540.1	Hu et al. (2023)
		*Rhinogobius duospilus*	MH127918.1	Tan et al. (2020)
		*Rhinogobius filamentosus*	OM678440.1	Chen et al. (2022)
		*Rhinogobius wuyanlingensis*	OM961051.1	Shang et al. (2022)
		*Rhinogobius estrellae*	LC648293.1	Maeda et al. (2021)
		*Rhinogobius tandikan*	LC648297.1	Maeda et al. (2021)
		*Rhinogobius sp. HN*	ON411594.1	–
		*Rhinogobius similis*	LC648304.1	Maeda et al. (2021)
	*Tridentiger*	*Tridentiger obscurus*	MF663787.1	Gong et al. (2017)

## 3. Results

### 3.1. Mitogenome organization

**Figure 2.**
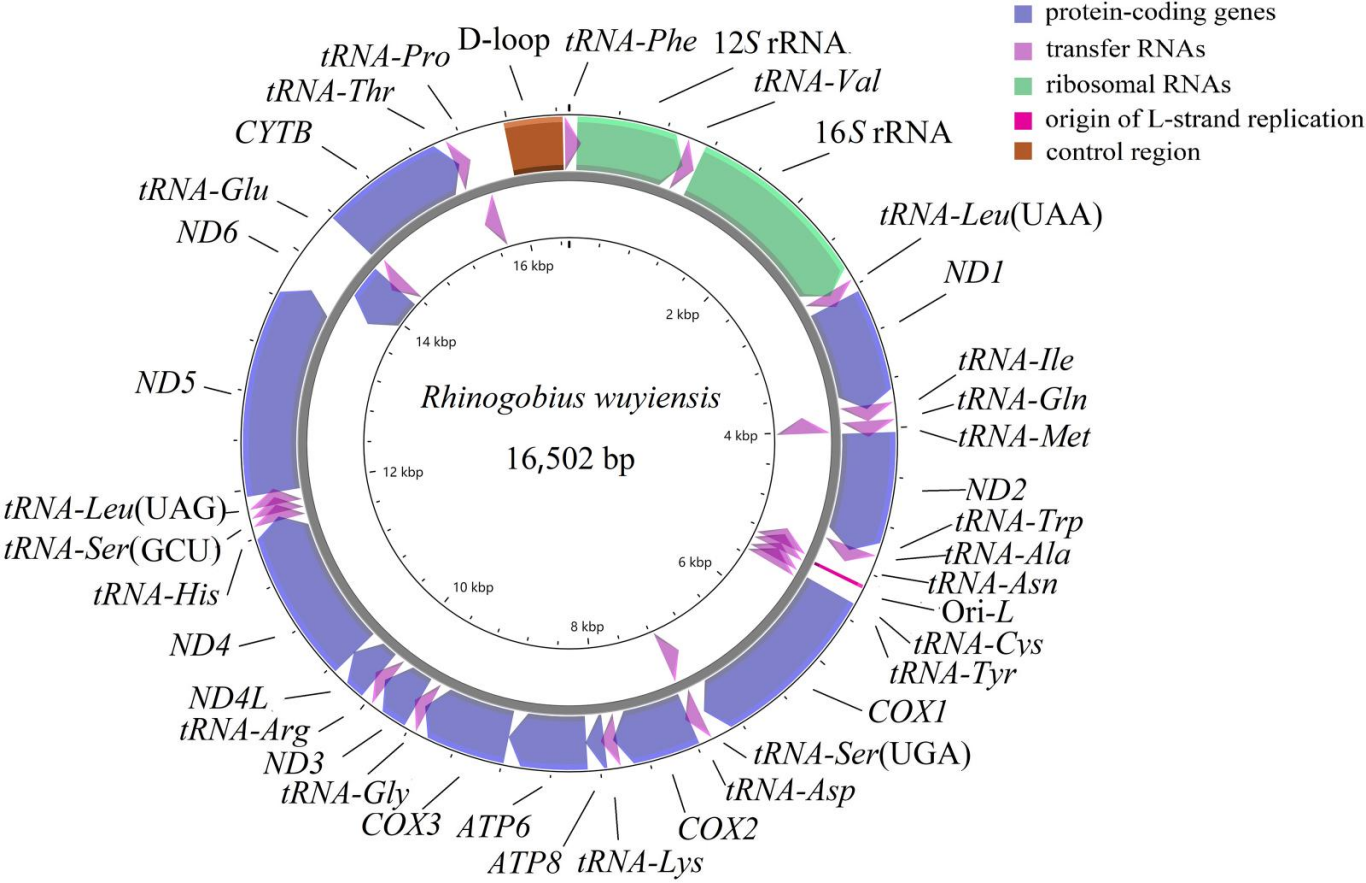
The gene map of the mitochondrial genome of *R. wuyiensis* illustrates the positioning of genes on both the heavy and light strands, with arrows indicating their transcriptional orientation. Genes encoded on the heavy strand are portrayed outside the circle, whereas those on the light strand are depicted inside.

The final circular genomic sequence of *R. wuyiensis* was determined to be 16,502 base pairs (bp) (GenBank Accession No. OM678441). It comprises 13 PCGs, 22 transfer RNA (tRNA) genes, two ribosomal RNA (rRNA) genes, and a control region (Figure 2). Most genes are encoded on the heavy strand (H-strand), except for the *ND6* gene and eight tRNA genes (*tRNA-Ser (UGA)*, *tRNA-Tyr*, *tRNA-Cys*, *tRNA-Asn*, *tRNA-Ala*, *tRNA-Gln*, *tRNA-Pro*, and *tRNA-Glu*). The overall nucleotide composition is 27.5% adenine (A), 24.9% thymine (T) (uracil (U) in RNA), 30.7% cytosine (C), and 16.9% guanine (G). Among the 13 PCGs, only *COX1* initiates with the codon GTG, while the remaining 12 genes commence with the canonical ATG codon. Eight PCGs (*ND1*, *ND2*, *COX1*, *ATP8*, *ATP6*, *ND4L*, *ND5*, and *ND6*) terminate with complete TAA or TAG codons, whereas five genes (*COX2*, *ND3, ND4*, *CYTB*, and *COX3*) contain incomplete termination codons (T or TA). The 22 tRNA genes adopt typical cloverleaf secondary structures and range in size from 66 to 76 bp. The control region spans 479 bp and is located between the *tRNA-Phe* and *tRNA-Pro* genes (Figure 2). The 13 PCGs range in length from 165 bp to 1839 bp. The two rRNA genes include a 12*S* rRNA (952 bp) and a 16*S* rRNA (1642 bp).

### 3.2. Phylogenetic analysis

**Figure 3.**
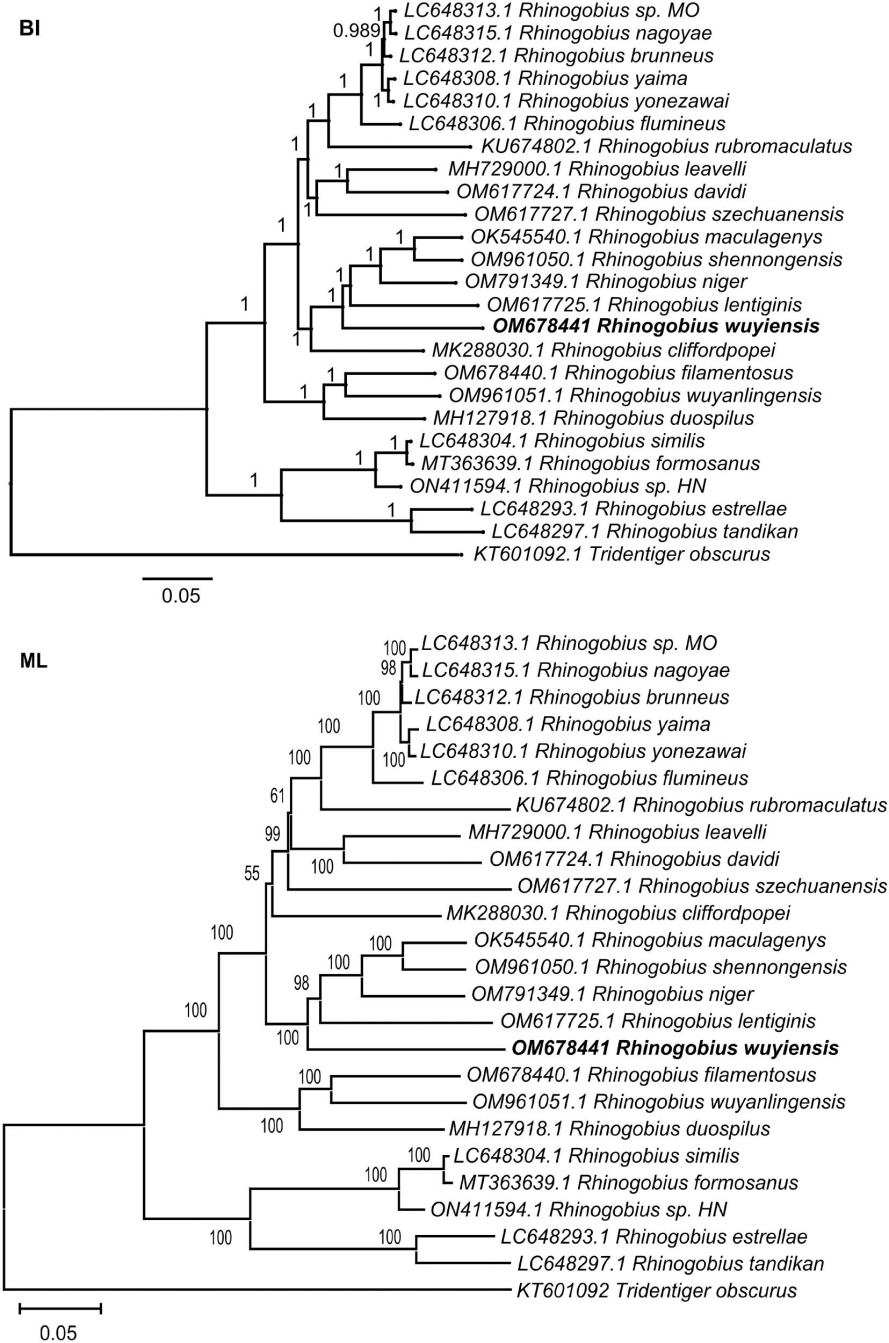
BI and ML phylogenetic trees were generated using datasets that included nucleotide sequences of all 13 protein-coding genes from *R. wuyiensis* and 23 additional species within the genus *Rhinogobius*. MT363639 (Yang et al. 2020), LC648316\LC648313\LC648312\LC648308\LC648310\LC648306\LC648293\ LC648297\LC648304 (Maeda et al. 2021), OM617727 (Liu et al. 2023), MH729000 (Zhang and Shen 2019), OM617724 (Song et al. 2023), MK288030 (Chen et al. 2019), OM617725 (Song et al. 2023), OM961050 (Shang et al. 2022), OK545540 (Hu et al. 2023), MH127918 (Tan et al. 2020), OM678440 (Chen et al. 2022), OM961051 (Shang et al. 2022), and KU376498 (Gong et al. 2017). Bootstrap support values (ML, based on 1000 replicates) and Bayesian’s posterior probabilities were annotated above branches. Each species is accompanied by its respective GenBank accession number.

The topology of the phylogenetic tree, reconstructed using both BI and ML methods, was consistent across both analyses, revealing two primary clades within the genus *Rhinogobius* (Figure 3). One clade comprised *R. similis*, *R. formosanus*, *R. sp. HN*, *R. estrellae*, and *R. tandikan*, while the other clade included 19 additional *Rhinogobius* species. Our findings differ from those reported by Song et al. (2023), who reported *R. estrellae* and *R. tandikan* as forming a single clade, with the remaining 21 *Rhinogobius* species in another clade. This discrepancy may stem from differences in the datasets used, including variations in GenBank accession numbers for potentially identical species, indicating that some taxa may not be conspecific. Both of our phylogenetic trees demonstrated that *R. wuyiensis* is closely related to a sister group comprising *R. maculagenys*, *R. shennongensis*, *R. niger*, and *R. lentiginis*, a result that is consistent with the findings of Song et al. (2023). The genetic distance between *R. wuyiensis* and the 23 other *Rhinogobius* species ranged from 0.12695 to 0.18670, while the distance between *R. wuyiensis* and its sister group ranged from 0.12695 to 0.13465.

## 4. Discussion and conclusions

In this study, we present the first complete mitochondrial genome of *R. wuyiensis*, analyze its structural characteristics, and infer its phylogenetic relationships based on the concatenated nucleotide sequences of 13 PCGs. The complete circular genome of *R. wuyiensis* comprises 16,502 bp, exhibits a gene order consistent with other members of the Gobionellinae subfamily (Song et al. 2023). A comparative analysis of sequences available on GenBank reveals variations in the control region length within the *Rhinogobius* genus. Specifically, *R. rubromaculatus* (KU674802) has a control region of 843 bp, while *R. wuyanlingensis* (OM617722) has a control region of 475 bp (Song et al. 2022). In contrast, the control region of *R. wuyiensis* measures 479 bp, underscoring the diversity in control region structure and organization among *Rhinogobius* species.

Phylogenetic analyses using BI and ML methods reveal that *R. wuyiensis* is closely related to a clade comprising *R. maculagenys*, *R. shennongensis*, *R. niger*, and *R. lentiginis*, further clarifying its taxonomic position within the genus *Rhinogobius*.

The mitochondrial genome data obtained from *R. wuyiensis* are anticipated to provide valuable insights for genetic research across various fields, including evolution, taxonomy, DNA barcoding, conservation biology, and phylogenetics.

## Supplementary Material

Supplementary Material.docx

## Data Availability

The genome sequence data that support the findings of this study are openly available in GenBank of NCBI at https://www.ncbi.nlm.nih.gov/ under the reference number OM678441. The associated ‘BioProject’, ‘Bio-Sample’, and ‘SRA’ numbers are PRJNA808170, SAMN26030914, and SRR18131291, respectively.
