# Supplementary material for: Characterization and phylogenetic analysis of the mitochondrial genome of Rhinogobius wuyiensis Li & Zhong, 2007 (Gobiiformes: Gobiidae: Gobionellinae)
Source: Mitochondrial DNA B Resour. 2025 Feb 18;10(3):233–8. doi: 10.1080/23802359.2025.2466584 (PMC11837909; doi:10.1080/23802359.2025.2466584)
Supplement: Supplementary Material.docx [file TMDN_A_2466584_SM3221.docx]

Supplementary Material for “Mitogenome characterization and phylogeny of *Rhinogobius wuyiensis* (Gobiiformes: [Gobiidae](https://www.ncbi.nlm.nih.gov/Taxonomy/Browser/wwwtax.cgi?mode=Undef&id=8220&lvl=3&keep=1&srchmode=1&unlock): Gobionellinae) ”


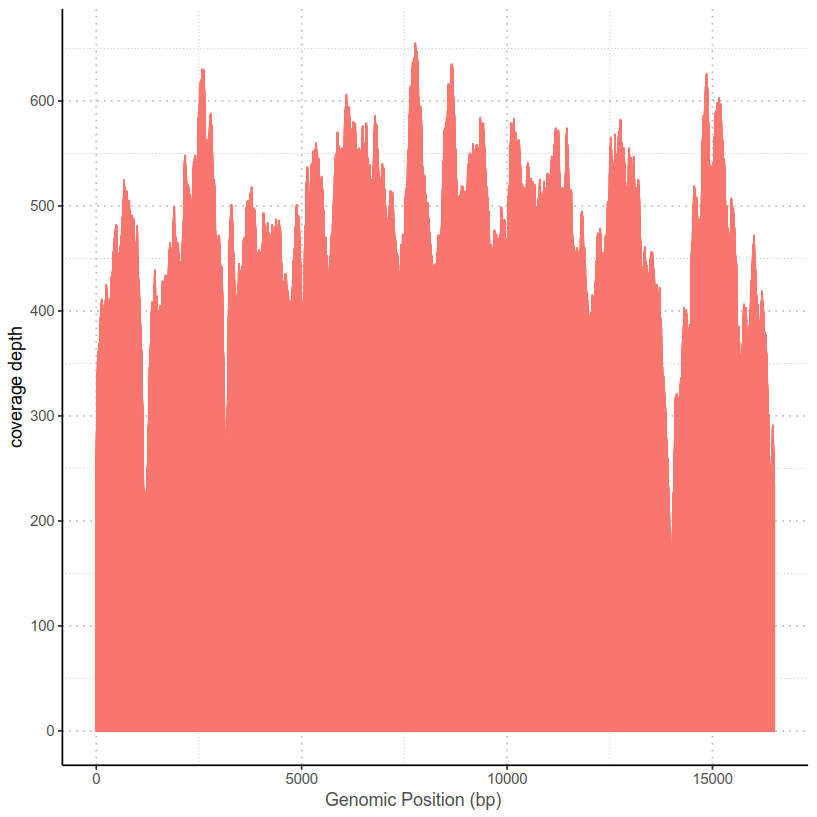


Figure 1. Coverage depth of the genome of *Rhinogobius wuyiensis*

| Sample | Contig Count | Total length | N50 | N90 | Min length | Max length |
| --- | --- | --- | --- | --- | --- | --- |
| *Rhinogobius_wuyiensis* | 459 | 332661 | 666 | 518 | 500 | 16629 |

| Sample | Reference | Reference  length(bp) | Contigs | Contigs  length(bp) | Ns(bp) | Ratio | GC% | Circular |
| --- | --- | --- | --- | --- | --- | --- | --- | --- |
| *Rhinogobius wuyiensis* | LC648312 | 16500 | 1 | 16502 | 0 | 0.00% | 47.60% | Yes |


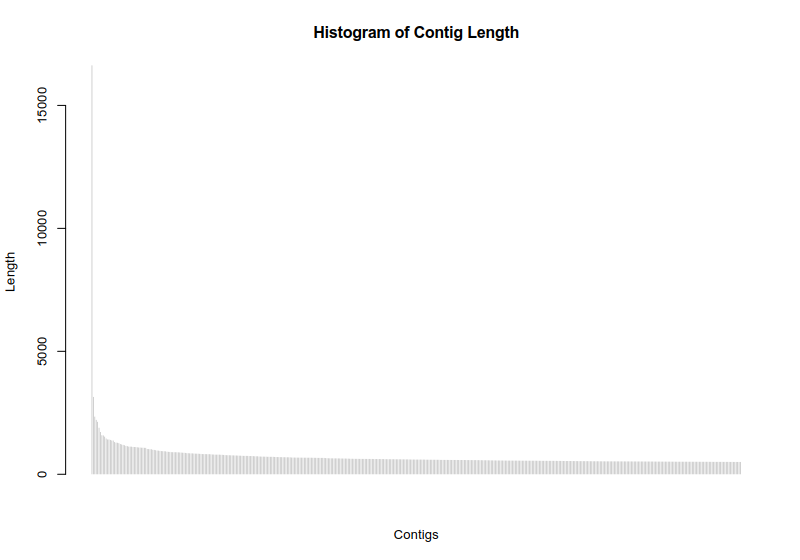


**Contig length distribution**

| # METRICS | *Rhinogobius wuyiensis* |
| --- | --- |
| BAIT_SET | Genbank annotation |
| GENOME_SIZE | 16500 |
| BAIT_TERRITORY | 16500 |
| TARGET_TERRITORY | 16500 |
| BAIT_DESIGN_EFFICIENCY | 1 |
| TOTAL_READS | 517928 |
| PF_READS | 517928 |
| PF_UNIQUE_READS | 517928 |
| PCT_PF_READS | 1 |
| PCT_PF_UQ_READS | 1 |
| PF_UQ_READS_ALIGNED | 14849 |
| PCT_PF_UQ_READS_ALIGNED | 0.02867 |
| PF_BASES_ALIGNED | 1712523 |
| PF_UQ_BASES_ALIGNED | 1712523 |
| ON_BAIT_BASES | 1712523 |
| NEAR_BAIT_BASES | 0 |
| OFF_BAIT_BASES | 0 |
| ON_TARGET_BASES | 1638939 |
| PCT_SELECTED_BASES | 1 |
| PCT_OFF_BAIT | 0 |
| ON_BAIT_VS_SELECTED | 1 |
| MEAN_BAIT_COVERAGE | 103.789273 |
| MEAN_TARGET_COVERAGE | 99.329636 |
| MEDIAN_TARGET_COVERAGE | 3 |
| MAX_TARGET_COVERAGE | 491 |
| PCT_USABLE_BASES_ON_BAIT | 0.022386 |
| PCT_USABLE_BASES_ON_TARGET | 0.021424 |
| FOLD_ENRICHMENT | 1 |
| ZERO_CVG_TARGETS_PCT | 0 |
| PCT_EXC_DUPE | 0 |
| PCT_EXC_MAPQ | 0 |
| PCT_EXC_BASEQ | 0.000018 |
| PCT_EXC_OVERLAP | 0.042978 |
| PCT_EXC_OFF_TARGET | 0.948501 |
| FOLD_80_BASE_PENALTY | ? |
| PCT_TARGET_BASES_1X | 0.52103 |
| PCT_TARGET_BASES_2X | 0.507879 |
| PCT_TARGET_BASES_10X | 0.481455 |
| PCT_TARGET_BASES_20X | 0.468485 |
| PCT_TARGET_BASES_30X | 0.450182 |
| PCT_TARGET_BASES_40X | 0.436 |
| PCT_TARGET_BASES_50X | 0.418 |
| PCT_TARGET_BASES_100X | 0.365273 |
| HS_LIBRARY_SIZE |  |
| HS_PENALTY_10X | 0 |
| HS_PENALTY_20X | 0 |
| HS_PENALTY_30X | 0 |
| HS_PENALTY_40X | 0 |
| HS_PENALTY_50X | 0 |
| HS_PENALTY_100X | 0 |
| AT_DROPOUT | 0 |
| GC_DROPOUT | 0 |
| HET_SNP_SENSITIVITY | 0.506654 |
| HET_SNP_Q | 3 |
